# Quantitative 3D seismic attribute analysis and probabilistic workflow for de-risking Badenian carbonate buildups in the Pannonian Basin, Hungary

**DOI:** 10.1038/s41598-026-50359-8

**Published:** 2026-04-27

**Authors:** Mohamed Ayed Elbalawy, Felicitász Velledits

**Affiliations:** 1 Faculty of Earth and Environmental Sciences and Engineering, Institute of Exploration Geosciences, University of Miskolc, Miskolc, Hungary; 2 Geophysics Department, Faculty of Science, Ain Shams University, Cairo, 11566, Egypt

**Keywords:** Carbonate buildups, 3D seismic attributes, Pannonian Basin, Badenian, Seismic geomorphology, Hydrocarbon exploration, Climate sciences, Solid Earth sciences

## Abstract

Carbonate buildups host nearly 40–60% of the world’s conventional hydrocarbon reserves, yet their recognition on seismic data remains difficult due to morphological similarity with volcanic edifices, erosional remnants, and tectonic highs. Existing workflows for isolated carbonate buildup (ICB) detection formalize criteria based on regional context, morphology, and internal geometry, but they were largely developed for long-lived, tectonically stable settings and are less effective in geologically complex basins. In the Pannonian Basin, carbonate growth was short-lived, syn-tectonic, and modified by rapid burial and diagenesis, producing smaller and less seismically expressive buildups. A modified, more quantitative approach is therefore required. We apply a refined ICB detection workflow to 3D seismic data from the Badenian (Middle Miocene) succession of the Pannonian Basin, Hungary. The modification eliminates non-essential criteria and introduces new diagnostics, including integrated seismic attributes (instantaneous amplitude, phase, pseudo-relief), crossline and inline consistency, and time-slice signatures. Horizon flattening, calibrated with well logs, cores, and calcareous nannoplankton biostratigraphy, enabled robust interpretation of carbonate morphology and platform evolution. Fifteen candidate ICBs were identified and ranked probabilistically: four probable (> 55%), six possible (45–55%), and five disregarded (< 45%), yielding a mean probability of success of 41% and approximately six high-potential prospects. Biostratigraphic evidence independently confirms a rapid drowning event, marked by subsidence-driven deepening from ~ 30 m to ~ 200 m and a transition to open-marine conditions. This attribute-driven, probabilistic workflow reduces interpretive subjectivity and offers a transferable methodology for predictive reservoir characterization in the Central Paratethys and comparable basins worldwide.

## Introduction

Carbonate buildups are among the most economically significant depositional systems, hosting an estimated 40–60% of the world’s conventional hydrocarbon reserves^1,2^ and deep geothermal reservoir potential^3^. Their high porosity, susceptibility to diagenetic enhancement, and tendency to form stratigraphic or structural traps make them prolific reservoirs, as demonstrated in the Permian Basin (USA), the Precaspian Basin (Kazakhstan), and the Central Luconia Province (offshore Sarawak)^4,5^. Yet, despite their importance, carbonate buildups remain among the most challenging exploration targets because of their heterogeneity and their seismic resemblance to non-carbonate features such as volcanic mounds, tilted fault blocks, and erosional remnants^1^.

The exploration risk associated with these ambiguities is well illustrated by a review of 60 wildcat wells drilled by Exxon between 1975 and 1987^6^. More than half of the wells (54%) failed to encounter carbonate buildups: 15.6% intersected erosional remnants, 12.5% penetrated non-carbonate lithologies, and 27.5% failed due to poor seismic data quality. Even where buildups were present, many proved non-commercial because of inadequate reservoir development, sealing failure, or lack of hydrocarbon charge. These results demonstrate that seismic recognition of isolated carbonate buildups (ICBs), while necessary, is not sufficient to ensure exploration success.

Seismic reflection data remains the primary tool for identifying ICBs. Beyond simple morphological recognition, seismic attribute analysis has become a cornerstone of stratigraphic interpretation. Attributes derived from complex trace analysis such as instantaneous amplitude, phase, and pseudo-relief can enhance lithological contrasts, delineate reflector terminations, and provide geomorphic visualizations that approximate outcrop perspectives^7,8^. When combined with horizon flattening, attributes enable reconstruction of paleobathymetric surfaces, a critical step in detecting shallow-water buildups^9^. Such approaches have proven especially effective in Southeast Asia, where laterally extensive Miocene carbonate platforms underpin prolific petroleum systems^10^.

A major milestone was the workflow of Burgess et al. 2013^10^, which shifted isolated carbonate buildup (ICB) recognition from largely intuitive, morphology-driven interpretation toward a structured and auditable sequence of tests. Rather than relying solely on mound-like geometry, the workflow requires candidate buildups to be evaluated using multiple independent seismic criteria, including regional stratigraphic context, external morphology, geophysical response, and internal reflector architecture. This multi-criteria logic improves discrimination between true carbonate buildups and common seismic mimics such as volcanic edifices, erosional remnants, and tectonic highs, while enhancing consistency among interpreters by making the basis for interpretation explicit. The workflow formalized this approach into a four-step interpretive sequence encompassing (i) regional and stratigraphic context, (ii) large-scale seismic morphology, (iii) geophysical characteristics, and (iv) finer-scale seismic geometries. However, it was developed primarily from Southeast Asian examples characterized by long-lived carbonate factories and relatively simple tectonic settings, and its direct application to structurally complex basins hosting short-lived carbonate systems remains limited.

The Pannonian Basin of Central Europe represents such a complex setting. Formed as a Miocene back-arc basin bounded by the Alps, Carpathians, and Dinarides, it records rapid extension and episodic marine incursions^11^. During the Badenian stage (~ 14.15 to 12.6 Ma), a major marine transgression flooded the Central Paratethys, creating transient shallow-marine environments favorable for carbonate growth^12–14^. The resulting buildups were isolated, fault-controlled, and embedded within siliciclastic successions, differing markedly from the vast, long-lived platforms of Southeast Asia. Their growth was short-lived (~ 1.5 My), syn-tectonic, and strongly modified by burial and diagenesis^13–15^

**Fig. 1:**
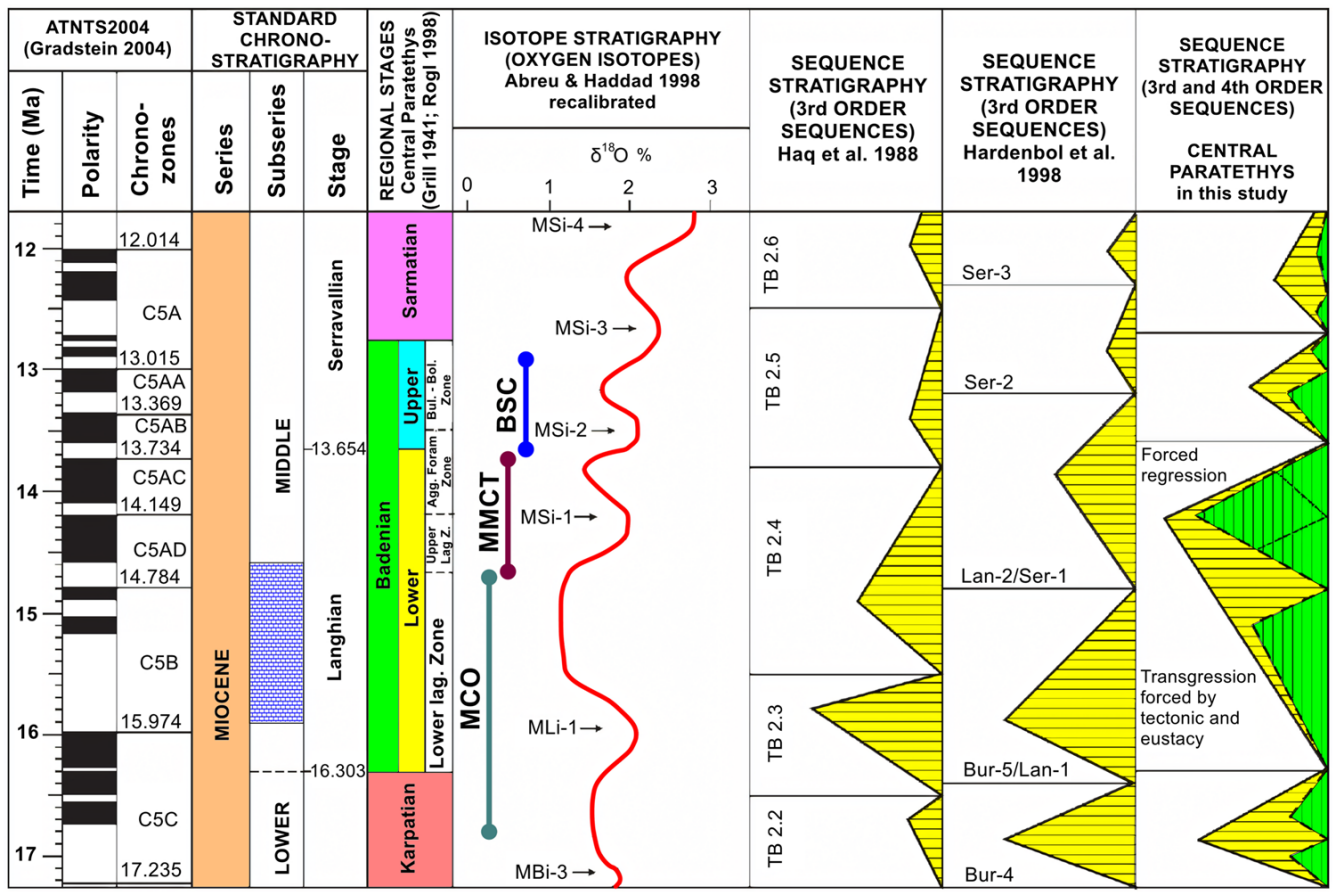
Integrated chronostratigraphic, isotope, and sequence stratigraphic framework of the Central Paratethys, highlighting Badenian third- and fourth-order cycles and their relationship to global isotope events. (*MCO* Miocene Climatic Optimum, *MMCT* Middle Miocene Climatic Transition, *BSC* Badenian Salinity Crisis).

Micropaleontological evidence underscores this complexity. Calcareous nannoplankton analyses from the Zala subbasin indicate a rapid drowning event within the NN5 zone (Upper Langhian zone; see Fig. 1), marking a shift from shallow-water carbonates to open-marine conditions. Such abrupt paleoenvironmental changes complicate stratigraphic correlation and can obscure the seismic expression of buildups.

Although sedimentological and paleontological studies of Badenian carbonates exist^15,16^, no integrated, quantitative seismic workflow has systematically addressed their identification or exploration potential. This study addresses that gap by applying a modified Burgess workflow to a new 3D seismic dataset from the Zala subbasin, Hungary. The modification introduces quantitative attribute integration, horizon flattening, and probabilistic classification calibrated with well-log and biostratigraphic data. Our approach reduces interpretive subjectivity, enables probabilistic ranking of candidate buildups, and provides a transferable methodology for de-risking carbonate exploration in frontier and structurally complex basins.

## Material and methods

### Study area

**Fig. 2:**
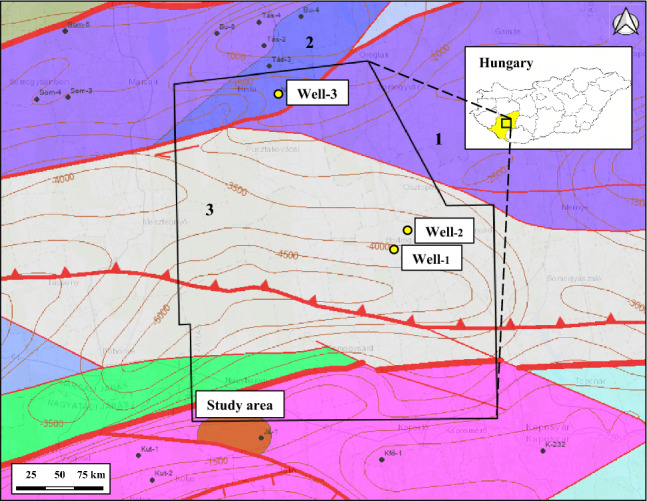
Geological map of the Pre-Tertiary basement (after^11^) showing the location of the study area and outline of the 3D seismic with the available wells. 1. Middle–Upper Triassic formations of platform and basin facies, 2. Lower Triassic shallow marine claystone, marl, limestone, and 3. Basement. Contour lines represent depth in meters.

The study area is situated south of Lake Balaton, next to the Balaton tectonic zone and on the northeast (NE) margin of the Zala subbasin complex, where an older northeast-southwest (NE-SW) Triassic structural trend was detected (Fig. 2).

### Geological history and tectonic evolution

#### Regional tectonic setting

**Fig. 3:**
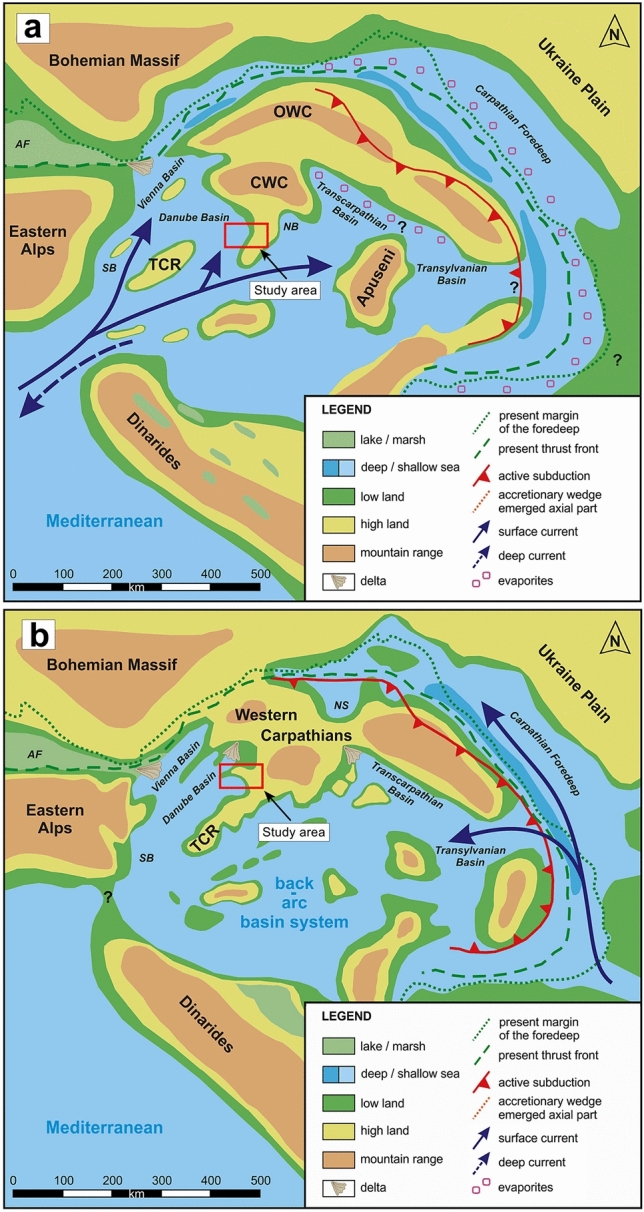
Palaeogeographic reconstructions of the Central Paratethys during the Early Middle Miocene to Late Middle Miocene (after^14^). **a** Early Middle Miocene. **b** Late Middle Miocene. Explanatory notes: (AF) Alpine Foredeep; (CWC) Central Western Carpathians; (NB) Novohrad-Nógrád Basin; (OWC) Outer Western Carpathians; (SB) Styrian Basin; (TCR) Transdanubian High; (?) assumed short-lived sea connection. the approximate location of the study area is indicated by the red box.

The Pannonian Basin is a major intramontane back-arc depression in Central Europe, bounded by the Alps, Carpathians, and Dinarides. Its geodynamic evolution was governed by Oligocene–Miocene rollback of the Carpathian slab, lithospheric thinning, and lateral extrusion of crustal blocks along major strike-slip fault systems^17–19^. Extension commenced in the Early Miocene (~ 20 Ma), producing rapid subsidence and a mosaic of half-graben depocentres, including the Zala, Danube, Drava, Vienna, and Styrian basins^13,20^ (see Fig. 3). Syn-sedimentary faulting strongly influenced accommodation, sediment routing, and the localization of carbonate factories on fault-controlled highs. Later compressional inversion and strike-slip reactivation overprinted these rift structures, generating a complex tectono-stratigraphic architecture that complicates the seismic expression of Miocene carbonate buildups^19^.

#### Stratigraphic framework

The Central Paratethys was a semi-isolated epicontinental sea whose depositional history records the interplay of global eustatic change, tectonic subsidence, and fluctuating connections with the Mediterranean and Eastern Paratethys^12–14^. Regional stratigraphy is subdivided into the Ottnangian, Karpatian, Badenian, Sarmatian, and Pannonian stages, calibrated through integrated biostratigraphy, magnetostratigraphy, radiometric dating, and astronomical tuning^21^.

The Badenian (~ 16.3 to 12.6 Ma) represents the most critical interval in the Miocene evolution of the basin. A major marine transgression re-established connections with the Mediterranean via the Alpine and Dinaridic gateways^12,21^, initiating shallow-marine carbonate production across structural highs. Carbonate deposition was short-lived, however, and rapidly terminated by drowning, followed by marl and evaporite accumulation.

Sequence-stratigraphic correlations (Fig. 1) compare global eustatic reference curves with a regionally calibrated framework for the Central Paratethys, illustrating how Badenian sedimentation reflects global forcing modified by local controls. The global third-order sea-level curves (Haq et al., 1998 and Hardenbol et al., 1998 Sequence-stratigraphic columns) represent worldwide eustatic fluctuations, whereas the Central Paratethys in this study column shows how these signals are locally expressed as relative sea-level changes. Badenian sedimentation was primarily governed by global third-order cycles (highlighted in yellow), overprinted by higher-frequency fourth-order oscillations (green) that are characteristic of the Central Paratethys^22,23^.

The Badenian stratigraphic architecture is therefore influenced by both global sea-level variations and localized processes, most notably syn-tectonic subsidence, basin segmentation, and sediment input. As a result, one to three third-order relative sea-level cycles may be identified across different Central Paratethys sub-basins, and direct correlation with global curves (e.g.,^22,23^) is not always straightforward due to strong regional overprinting. Oxygen-isotope stratigraphy records cooling pulses (MSi-1 to MSi-4) that intermittently disrupted carbonate productivity^24^. Key stratigraphic surfaces, including the Bur5/Lan1 transgressive pulse and the Lan2/Ser1 forced regression, further illustrate the combined influence of eustatic forcing and tectonic subsidence on Badenian sedimentation.

Climatic records indicate a stepwise trend from warm–humid subtropical forests in the early Miocene to cooler, more arid conditions by the Late Badenian–Sarmatian. Pollen spectra and macrofloras document the rise of xerophytic and halophytic taxa, while sedimentological evidence points to alternating estuarine and anti-estuarine circulation regimes that regulated salinity, oxygenation, and carbonate productivity^21,25^.

Thus, the Badenian represents a pivotal turning point: an interval of prolific shallow-marine carbonate deposition during the MMCO, followed by abrupt drowning, evaporite crises, and progressive isolation of the Central Paratethys system.

#### Palaeogeography and palaeoclimate evolution

Palaeogeographic reconstructions (Fig. 3a, b) highlight the dynamic evolution of the Central Paratethys from open-marine to progressively restricted conditions and showing shifting gateways, circulation regimes, and depositional environments. During the Early Badenian (Langhian; Fig. 3a), extensive marine flooding established broad carbonate shelves and archipelagos. Open connections with the Mediterranean enabled faunal exchange and supported prolific carbonate factories dominated by coralline algae and larger benthic foraminifera, with reefal corals playing only a minor role^15^.

The Badenian coincided with the Mid-Miocene Climatic Optimum (MMCO), when warm-temperate to subtropical conditions supported diverse carbonate and molluscan assemblages^13,26,27^. However, stable isotope curves (Fig. 1) document short-lived cooling events (MSi-2, MSi-3) that reduce carbonate growth. By the Late Badenian (early Serravallian; Fig. 3b), cooling, tectonic restriction, and elevated evaporation drove the transition to deeper-water marls, evaporites, and brackish facies. This culminated in the Badenian Salinity Crisis (BSC) at ~ 13.8 Ma, during which widespread evaporite basins developed in the Carpathian Foredeep, Transylvanian, and Transcarpathian domains^28^.

## Research approach framework

**Fig. 4:**
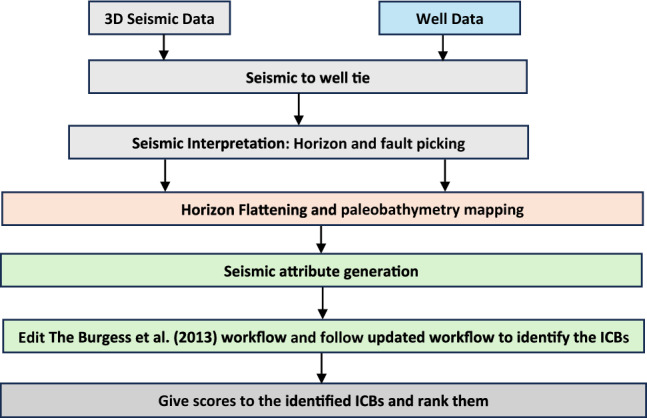
The applied workflow to identify the isolated carbonate buildups.

The dataset employed in this study integrates well, core, biostratigraphic, and 3D seismic data to constrain the depositional architecture and seismic expression of Badenian isolated carbonate buildups (ICBs) in the Zala Subbasin. Figure 4 displays the integrated workflow used in this study.

### Well data

**Fig. 5:**
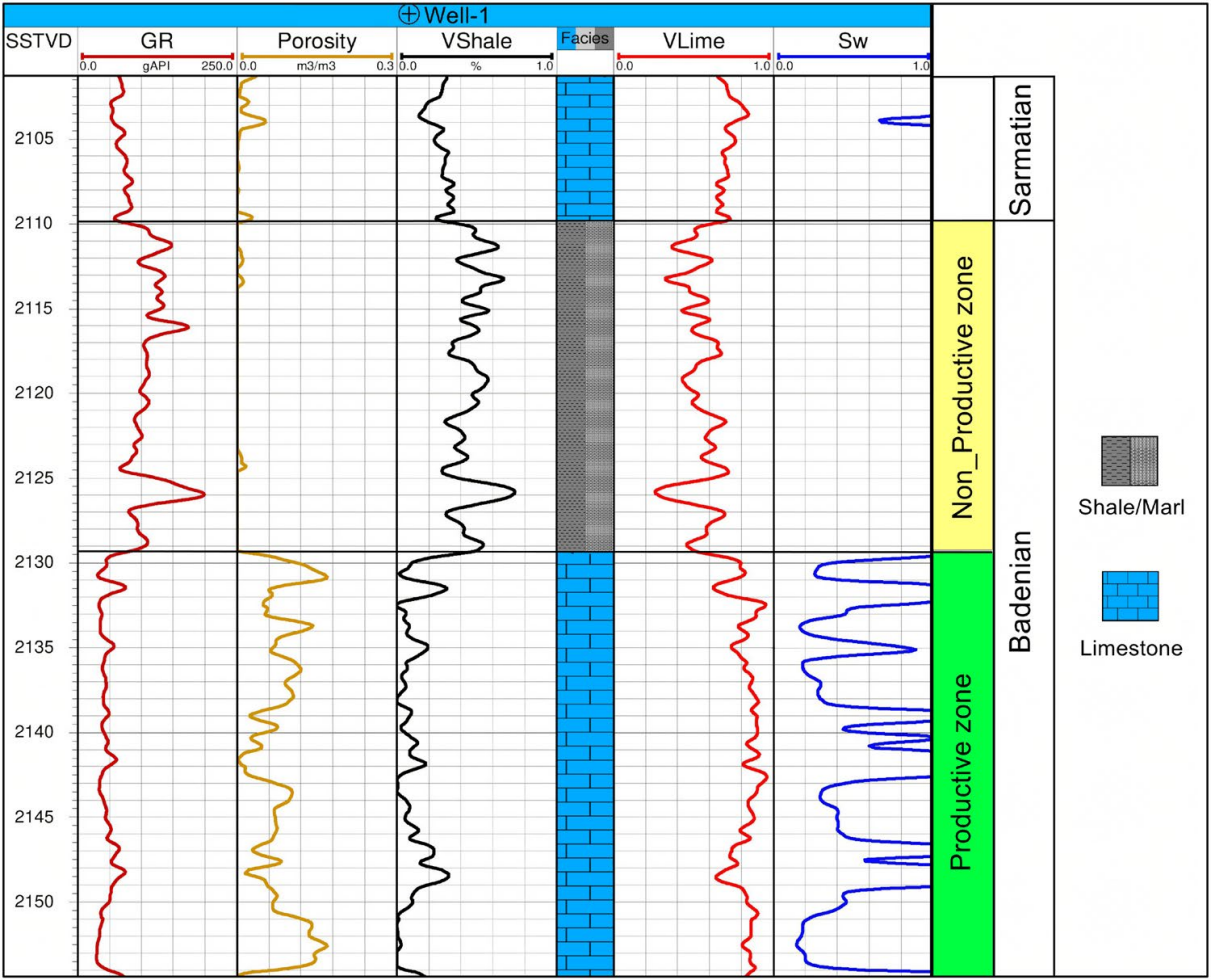
Well log display of well-1 showing the petrophysical properties of the Badenian productive and non-productive zones. *GR* gamma ray, *Vshale* volume of shale, *Vlime* volume of limestone, and *Sw* water saturation.

Three exploration wells supplied a comprehensive suite of wireline logs, including gamma ray, resistivity, density, sonic, neutron porosity, and caliper measurements, complemented by core descriptions that documented lithological textures, facies, and diagenetic features. Petrophysical analysis of gamma ray logs enabled discrimination between productive lithothamnium limestones, characterized by low gamma values (~ 50 API), and non-reservoir marls with high values (~ 110 to 120 API) (Fig. 5). Sonic–density calibration constrained seismic vertical resolution to approximately 30 m, thereby defining the detectability of carbonate units within the 3D seismic volume. Biostratigraphic analyses of calcareous nannoplankton refined the depositional age to the Badenian (NN5) and provided palaeoenvironmental information on fluctuations in temperature, salinity, and productivity, while foraminiferal assemblages indicated a rapid drowning event linked to subsidence.

### Seismic data

We used a 3D post-stack, time-migrated seismic dataset (SEG-Y format), acquired in 2019 and processed in 2020. The survey was recorded with an inline and crossline bin size of 25 m × 25 m and a 2 ms sample interval, using SEG positive polarity. Processing included pre-stack conditioning, surface-consistent amplitude corrections, deconvolution, multiple attenuation, statics correction, and pre-stack time migration (PSTM) based on the processing report. The dominant frequency within the target interval is approximately 40 Hz and 4500 m/s velocity, corresponding to an estimated vertical resolution of 28 m and a horizontal resolution of 56 m.

The post-stack seismic data was interpreted using the OpendTect software which provided tools for horizon picking, attribute extraction, and horizon flattening. The workflow began with conventional seismic interpretation to establish the structural and stratigraphic framework of the study area. The top Badenian horizon was selected as the key stratigraphic marker for carbonate buildup identification. This surface was mapped throughout the cube by manually picking continuous reflectors and interpolating across faulted segments. Major fault zones were interpreted in parallel, based on reflector terminations, offsets, and dip continuity.

### Seismic attribute extraction

Seismic attributes are mathematical transformations of seismic traces designed to highlight features not obvious in conventional amplitude displays^7^. For this study, three instantaneous attributes derived from complex trace analysis were selected: amplitude, phase, and pseudo-relief. Each attribute was extracted on the flattened top Badenian surface and analyzed in both vertical cross-sections and time slices.

#### Complex trace analysis

Seismic attributes derived from Hilbert transform–based complex trace analysis were applied to enhance carbonate buildup detection. In this approach, each seismic trace is represented as an analytic signal composed of the original trace and its quadrature (Hilbert-transformed) component, allowing the extraction of instantaneous attributes that describe the temporal behavior of seismic reflections independent of waveform polarity. Instantaneous amplitude (reflection strength) was used to highlight zones of strong impedance contrast commonly associated with carbonate margins, while instantaneous phase emphasizes reflector continuity and discontinuities that are diagnostic of buildup edges and internal architecture. The pseudo-relief attribute combines amplitude and phase information to generate outcrop-like visualizations that facilitate recognition of buildup morphology and seismic geomorphology. These attributes are widely used for stratigraphic interpretation and carbonate buildup detection, and their theoretical basis and practical applications are well established in the literature (e.g.,^7,29^).

### Horizon flattening and paleobathymetry

To restore depositional context, the top Badenian horizon was flattened to remove post-depositional tilting and burial effects. This approach generated paleobathymetry maps that highlight positive-relief carbonate buildups, syn-sedimentary fault scarps, and paleohighs controlling carbonate nucleation. The flattening horizon was carefully selected to meet three requirements: (1) It was flat at the time of deposition, (2) It lay as close as possible to the target interval, and (3) It covered the full extent of the study area. This reconstruction provided the most realistic approximation of Badenian paleomorphology and enabled delineation of depositional highs favorable for carbonate development.

### Carbonate buildup identification workflow

**Fig. 6:**
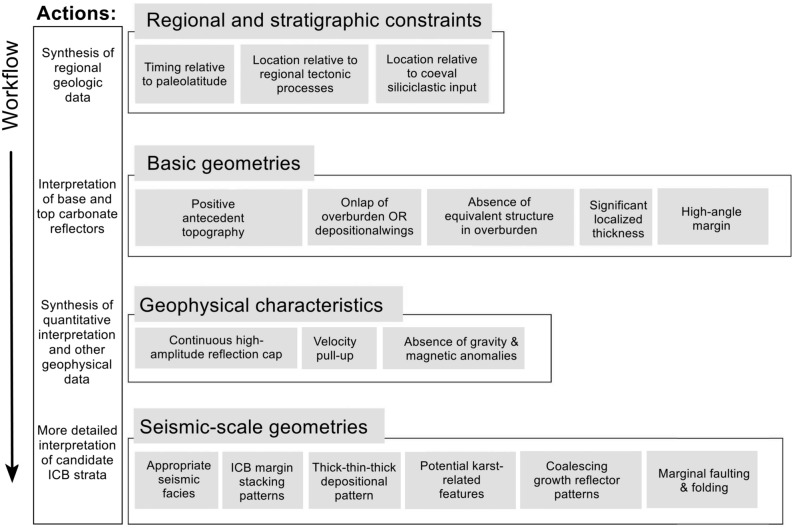
Flow chart indicating the sequence in which the identification criteria should be assessed. The arrow on the left indicates a progression of actions to be taken from synthesis of regional geologic data to analysis of fine-scale seismic geometries. For each scale of consideration, a series of criteria to assess working horizontally across the diagram from left to right exists^10^.

The detection of ICBs followed the criteria established by Burgess et al. (2013) (Fig. 6), who defined a four-step workflow comprising: (1) regional and stratigraphic context, evaluating paleogeographic setting, tectonic influences, and siliciclastic input; (2) basic morphologies, recognizing positive relief, isolated extent, and onlap geometries; (3) seismic-scale geometries, including margin stacking, reflector patterns, and karst-related features; and (4) geophysical characteristics, such as amplitude caps and velocity pull-up. While this framework has proven highly effective in Southeast Asian basins, its direct application to the Pannonian Basin proved problematic. Here, carbonate factories were smaller and shorter-lived, buildup morphologies were less pronounced, and a strong syn-tectonic overprint masked primary depositional relief.

**Fig. 7:**
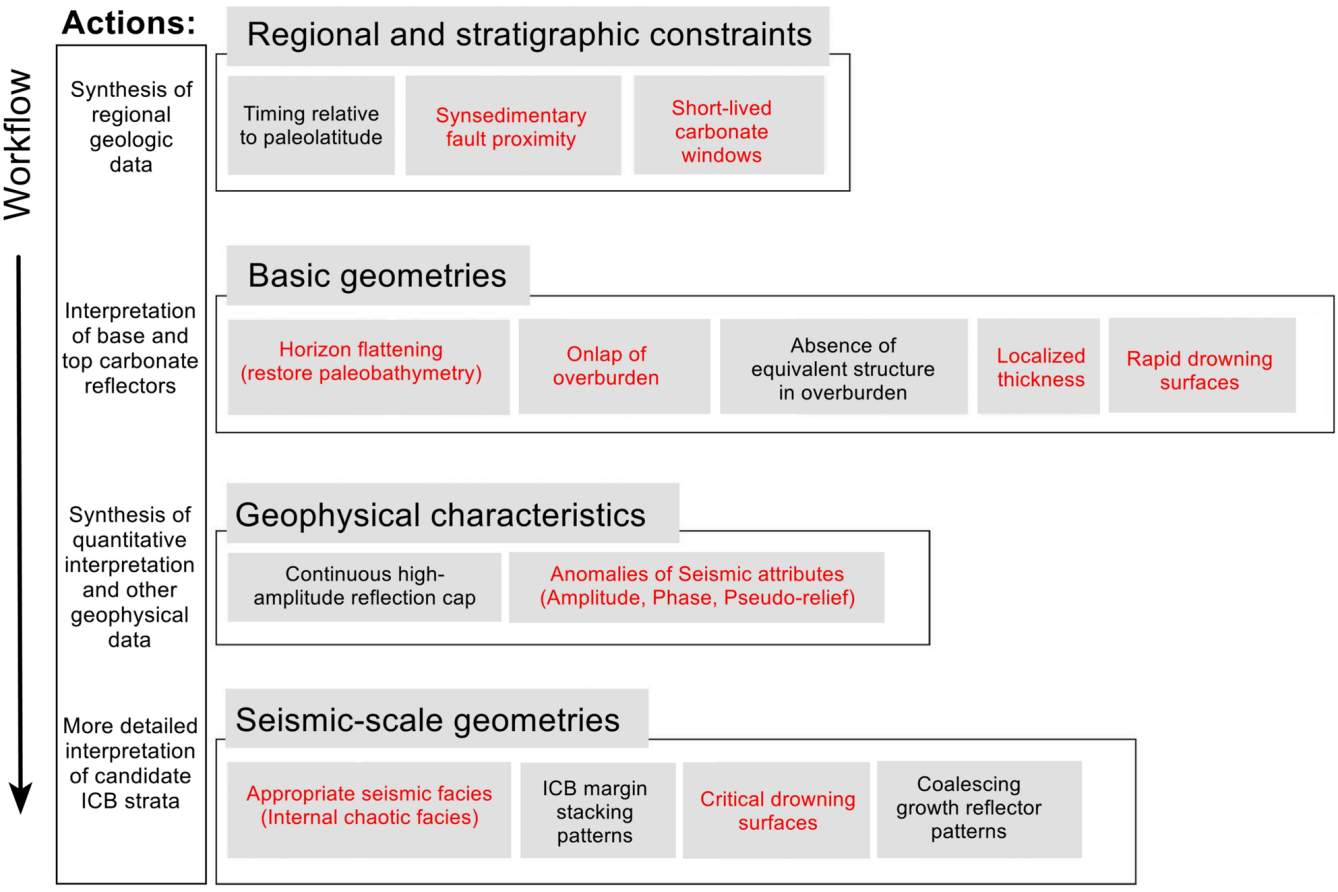
The modified workflow of Burgess et al., (2013).

To overcome these limitations, the workflow was adapted in two principal ways (Fig. 7). First, horizon flattening was integrated into the “Basic Geometries” step to restore paleobathymetry and reveal depositional relief otherwise obscured by structural tilting and differential subsidence. This step was essential in a syn-rift setting where carbonate nucleation commonly occurred along fault-block margins. Second, seismic attribute analysis was introduced within the “Geophysical Characteristics” step to enhance morphological and lithological detection. Instantaneous amplitude highlighted lithological contrasts and buildup margins, instantaneous phase improved recognition of reflector terminations and onlap geometries, and pseudo-relief provided geomorphic-style visualizations that emphasized mound morphologies in horizon slices. These attributes added a critical layer of interpretive confidence, especially where conventional reflection geometries alone were ambiguous.

Additional modifications included emphasizing syn-sedimentary fault proximity and short production windows under “Regional and Stratigraphic Constraints,” while de-emphasizing criteria such as velocity pull-up or gravity/magnetic anomalies, which are irrelevant in Badenian shallow-marine buildups. Within “Seismic-Scale Geometries,” internal chaotic facies zones and stratigraphic drowning surfaces were added to capture the effects of rapid burial and subsidence (Table 1).

**Table 1:** A comparison table between the original Burgess vs. Modified Pannonian workflow.

Burgess et al. (2013)	Modified Workflow (Pannonian Basin)	Add	Remove
Regional & Stratigraphic: palaeolatitude, tectonics, siliciclastic input	Syn-sedimentary fault proximity; short-lived carbonate windows	Emphasis on syn-sedimentary setting	Long-term stable factories
Basic Geometries: positive relief, localized thickening, onlap, isolated extent	Horizon flattening; rapid drowning surfaces	Restore paleobathymetry; drowning detection	Assumption of continuous growth
Geophysical Characteristics: high-amplitude cap, velocity pull-up, gravity/magnetic absence	Seismic attributes (Amplitude, Phase, Pseudo-relief)	Attribute-driven detection	Velocity pull-up; gravity & magnetic anomalies
Seismic-Scale Geometries: margin stacking, thick-thin-thick cycles, coalescing reflectors	Internal chaotic facies; drowning surfaces	Capture rapid burial and subsidence	Over-reliance on thick-thin-thick cycles

These modifications produced a Modified Burgess Workflow for Syn-Tectonic Basins, tailored to the Pannonian Basin, which integrates depositional relief restoration and attribute-driven quantification, thereby improving interpretive confidence in a structurally complex setting.

### Integration with well and core data

Well and core data were used to calibrate seismic interpretations of isolated carbonate buildups. Gamma ray logs differentiated carbonates (low values) from shaly marls (high values), while density and sonic logs enabled acoustic impedance calculations that highlighted carbonate contrasts. Porosity logs provided reservoir quality insights, and core descriptions confirmed lithology, depositional textures, and diagenetic overprint (Fig. 5). Calcareous nannoplankton zonation constrained the Badenian age of deposition. Seismic features intersected by wells were validated against carbonate lithology, whereas uncalibrated features were assigned probability scores to guide interpretive confidence.

## Results

### Well data analysis

**Fig. 8:**
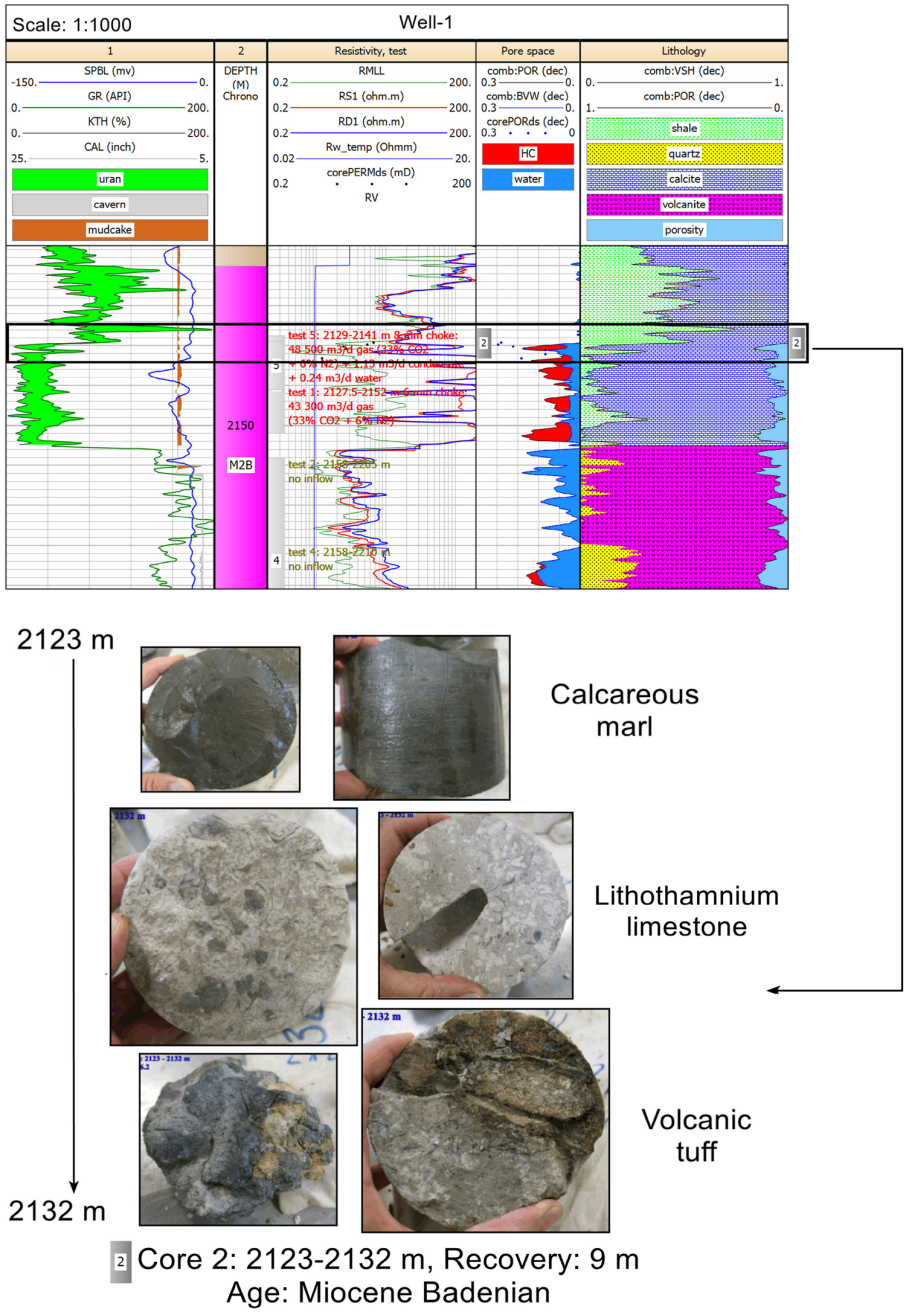
A petrophysical study conducted on well 1 (productive well). The core samples show the deposition of the deep marine fine grain calcareous marl and clay deposits on the top of the shallow water porous carbonates.

**Fig. 9:**
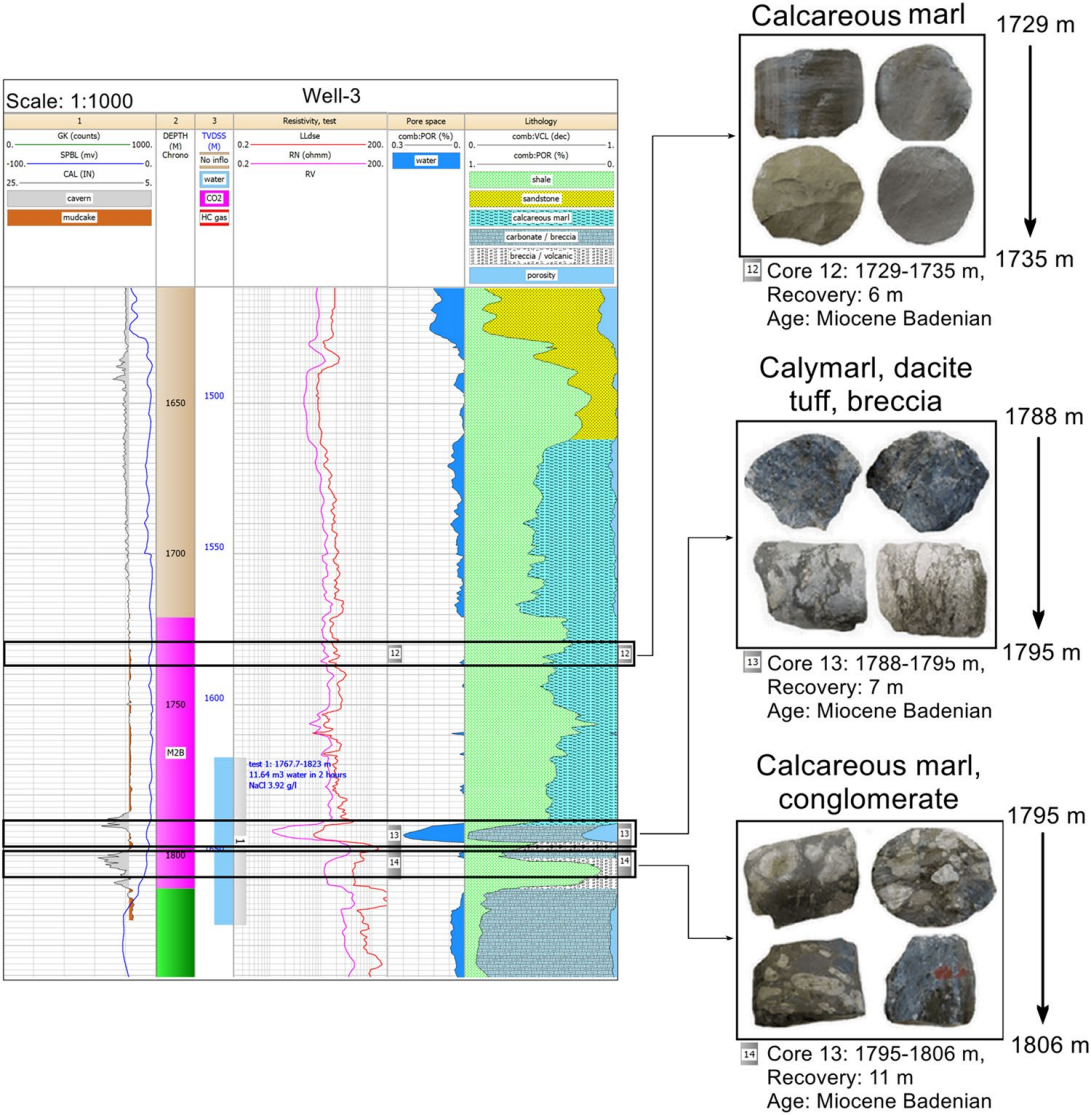
A petrophysical study conducted on well 3 (dry well). The core samples show the deepening event away from the carbonate platform indicated by deposition of deep marine fine grain calcareous marl and clay deposits.

To evaluate the control of Badenian carbonate sedimentology on productivity, we integrated wireline logs and core data from two wells, Well-1 which is productive and Well-3 which is dry. Figure 8 displays a petrophysical study conducted on well 1 (productive well). Note the dramatic increase in the gamma ray log reading (from around 50 API to 110 API) and the deposition of the deep marine fine grain calcareous marl and clay deposits on the top of the shallow water carbonates indicate the water flooding event due to the dramatic subsidence which is the main reason behind carbonate drowning. Figure 9 depicts a petrophysical study conducted on well 3 (dry well). Note the deposition of the deep marine fine grain calcareous marl and clay deposits on the top of the reworked carbonate clasts and the basinal coarse grain conglomerates. The core samples description confirms the deepening event away from the carbonate platform.

Micropalaeontological analysis (foraminifera) from the productive wells constrains a sharp relative sea-level increase at the limestone–marl boundary. Assemblages indicate deposition of the lower limestone in shallow water (≤ ~ 30 m), overlain abruptly by marls deposited in ~ 100 to 200 m. Because healthy carbonate factories typically keep pace with eustatic rise, this magnitude of deepening is best explained by tectonically driven subsidence/drowning during the Badenian.

Calcareous nannoplankton biostratigraphy of selected core samples (analysis by Stjepan Ćorić) yields an NN5 assignment, based on occurrences of *Coccolithus pelagicus*^30^, *Reticulofenestra perplexa*^31^, and *Reticulofenestra minuta*^32^. These data indicate platform drowning within NN5 and a shift to cooler, open-marine conditions.

Collectively, the log–core–seismic integration demonstrates that (i) productivity is confined to the low-GR Lithothamnium limestone, (ii) the overlying high-GR marls record rapid drowning, and (iii) the timing and palaeo-depth changes are consistent with a tectonic deepening event rather than eustasy alone.

### Seismic data interpretation

**Fig. 10:**
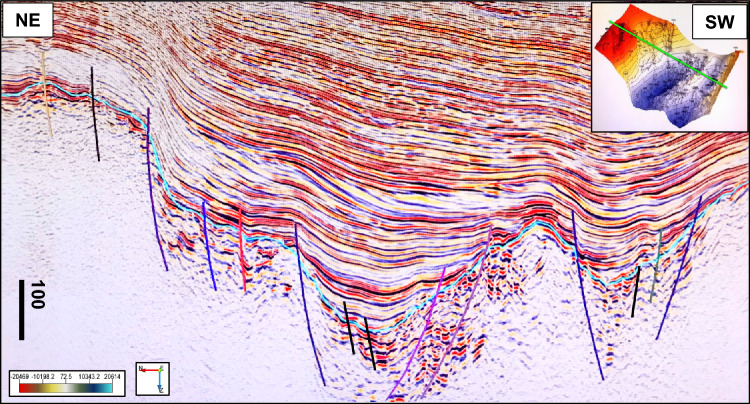
Structural interpretation marks out syn-rift faulting and related subsidence of separate depocentres with a location map on the upper right corner. Light blue layer: top Badenian.

The interpretation of the seismic data (Fig. 10) revealed that the present-day structure of the study area is strongly influenced by the geodynamic development of the Alpine-Carpathian Mountain chains and development the Pannonian Basin System and Carpathian Foredeep. Changes in the structural pattern (tectonics) of the area were highly influenced by subduction in front of the orogenies, as well as by the back-arc extension. The different driving forces, the changing geometry of the external Carpathian thrust system might have led to a spatially and temporally variable stress field^33^.

### Horizon flattening and paleogeographic context

**Fig. 11:**
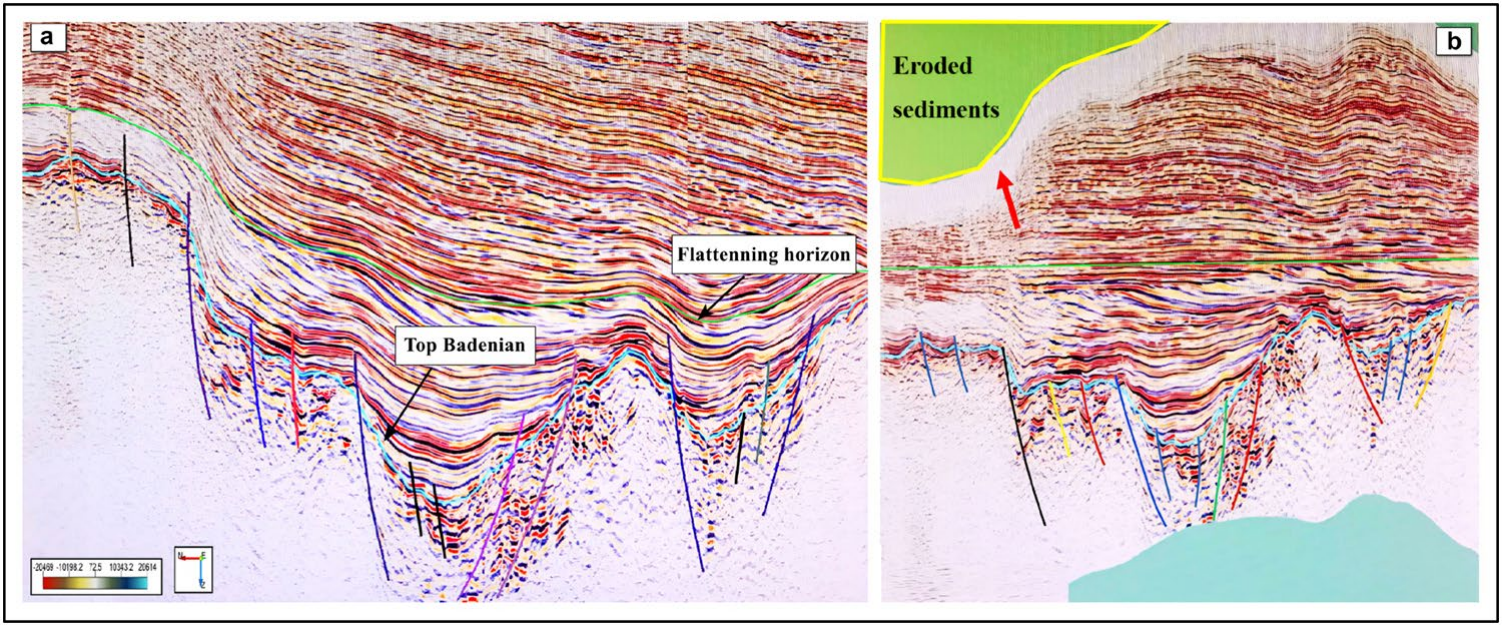
**a** Present-day structure of the study area where the green reflector indicates the flattening horizon and the light blue reflector depicts top Badenian. **b** Paleogeomorphology of the study area after flattening.

Flattening of the top Badenian horizon restored the depositional surface to a near-horizontal datum, enabling visualization of positive-relief features in their original geomorphic context. Figure 11 shows that nearly 500–600 m of sediments were eroded after flattening. Restoring the eroded sediments due to the basement uplift is key to generating reliable paleomorphology. In the unflattened cube, relief related to post-depositional tilting and fault block rotation often obscures subtle buildups. After flattening, at least fifteen discrete mounded features were evident, distributed across the central and northeastern portions of the seismic cube. The flattened surface revealed that many features preferentially nucleated adjacent to major syn-sedimentary fault scarps. These scarps provided localized bathymetric highs that served as ecological niches for reef initiation. Despite the seismic data resolution limitation and small size of the carbonate build ups, the flattening step enabled clear recognition of morphological contrasts.

### Seismic attributes

**Fig. 12:**
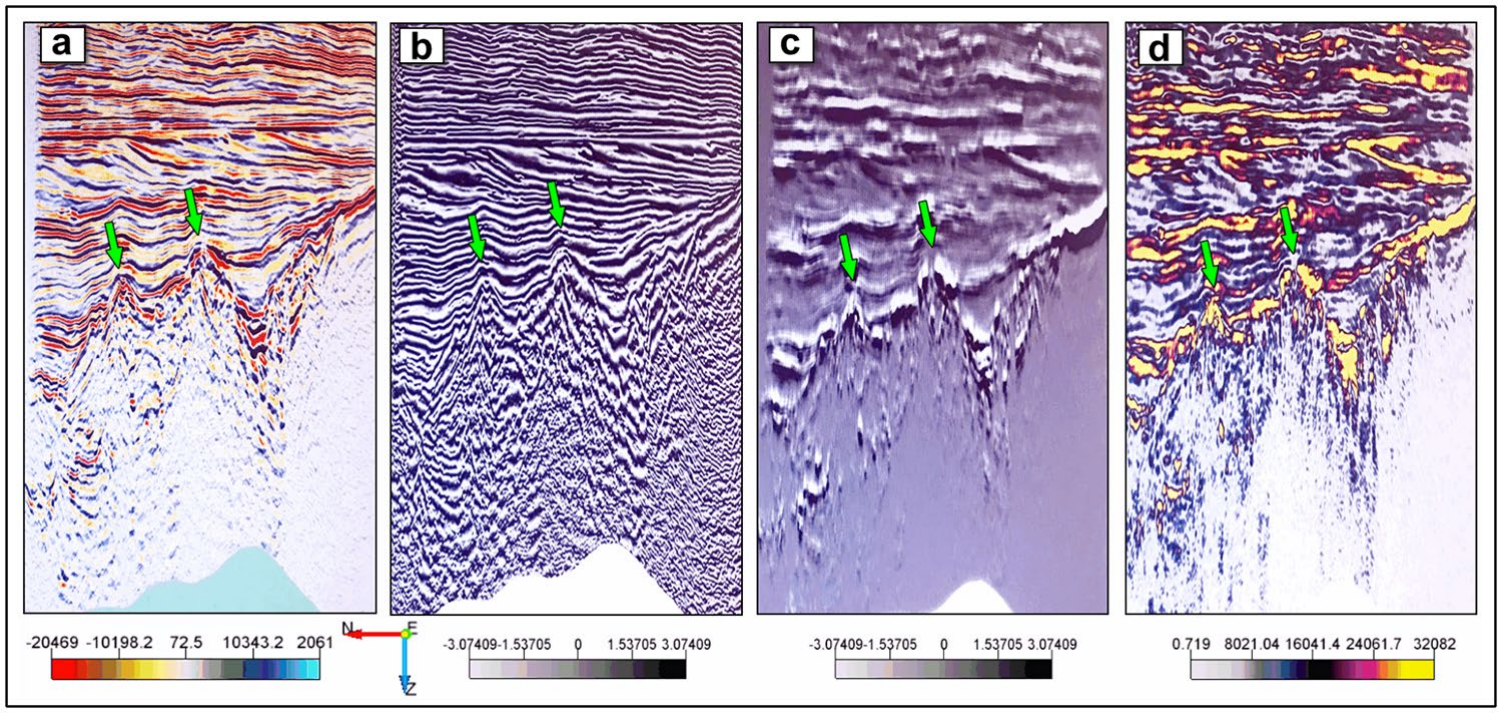
Seismic attribute results emphasizing the presence of carbonate features as indicated by the green arrow. **a** Full stack seismic, **b** Instantaneous Phase, **c** Pseudo Relief, and **d** Instantaneous Amplitude.

Attribute analysis proved crucial in differentiating true carbonate buildups from other positive-relief features. Figure 12 displays the seismic attribute results emphasizing the presence of the carbonate features.

#### Instantaneous phase

Phase attributes emphasize reflector continuity and discontinuity (Fig. 12b). Carbonate buildups were characterized by sharp phase discontinuities at their boundaries, where steep flanks caused reflector terminations. Internally, chaotic to subparallel phase patterns were observed, consistent with heterogeneous reef cores and lagoonal fills. Phase was particularly effective in highlighting subtle buildups of lower relief, where amplitude contrast alone was insufficient for confident interpretation.

#### Pseudo-relief

Pseudo-relief proved the most intuitive visualization tool. Buildups appeared as outcrop-like mounds, standing out against surrounding flat-lying marls (Fig. 12c). This attribute was especially useful for mapping morphology in map view, enabling comparison with analogs from exposed carbonate platforms.

#### Instantaneous amplitude

Amplitude slices highlighted carbonate margins as high-amplitude rims surrounding low-amplitude cores (Fig. 12d). This rimmed expression is diagnostic of reefal margins, where strong impedance contrasts develop between dense reefal frameworks and surrounding marls. Interiors often showed reduced amplitude, consistent with lagoonal or back-reef facies. Amplitude anomalies also allowed discrimination from erosional remnants, which typically displayed uniformly high amplitudes without rimmed geometry.

### Quantitative classification and ranking

The identification criteria were evaluated numerically by combining them into a composite score. To account for uncertainty in each diagnostic element, individual criteria were assigned the following values: + 1 for a definite positive response, + 0.5 for a weak positive response with some uncertainty, 0 when the criterion could not be reliably assessed (e.g., due to limited data quality), and − 1 for a definite negative response. The total score was calculated by summing the values of the 14 equally weighted criteria. The probability of success (PoS %) was then determined by normalizing the total score according to Eq. 1:

1 $$PoS(\mathrm{\%})=\left(\frac{\sum_{\mathrm{i}=1}^{14}{\mathrm{S}}_{\mathrm{i}}}{14}\right)\times 100$$

where, ${S}_{i}$: score assigned to each individual criterion and 14: total number of evaluation criteria.

Based on the resulting PoS values, the scoring scheme is defined as follows: (1) features with a probability of success (PoS) less than 45% are disregarded; (2) features with a PoS between 45 and 55% are classified as possible; and (3) features with a PoS greater than 55% are classified as probable.

**Fig. 13:**
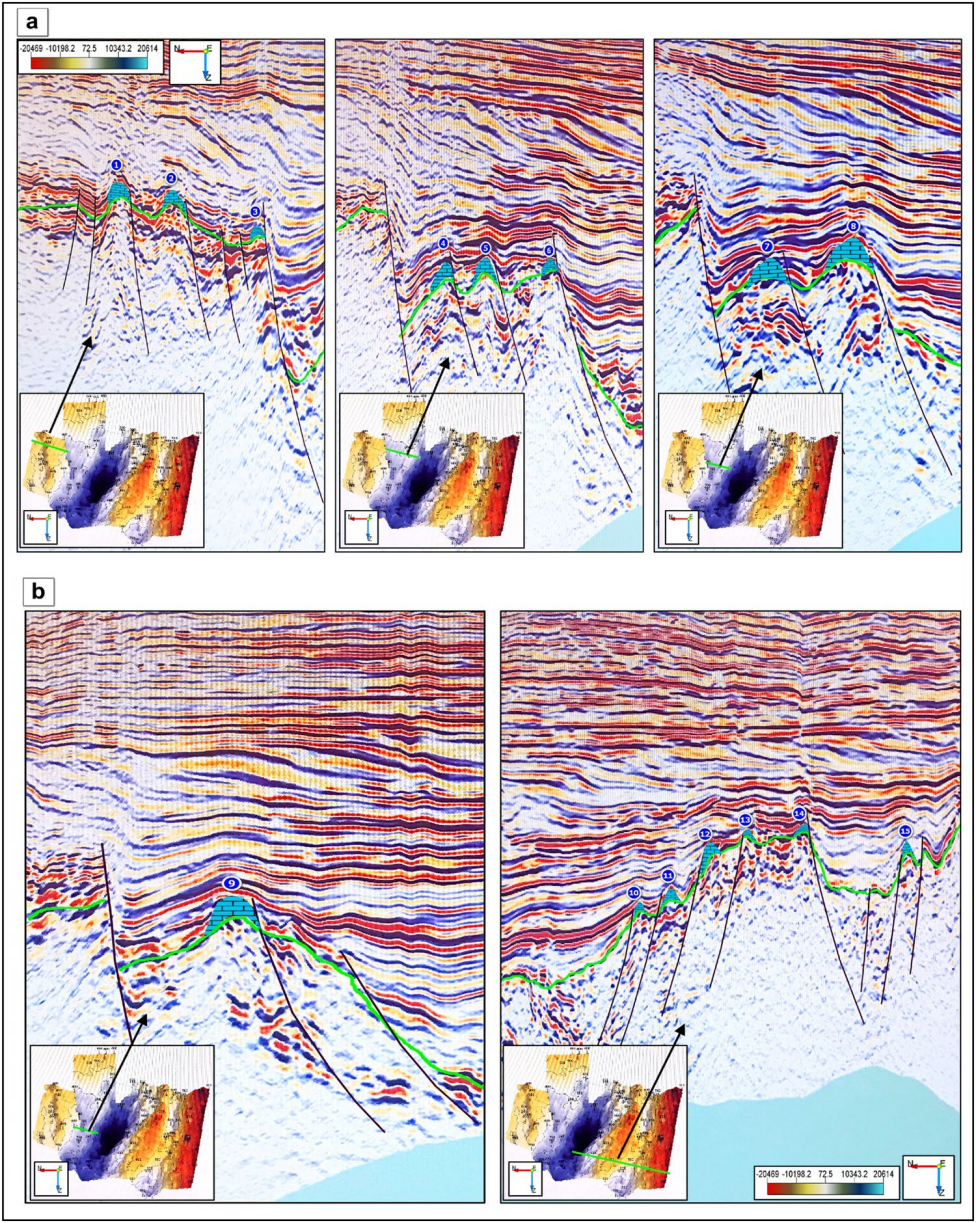
Identified carbonate features. **a** From number 1 to 8 imaged on the seismic data. **b** From number 9 to 15 imaged on the seismic data.

**Fig. 14:**
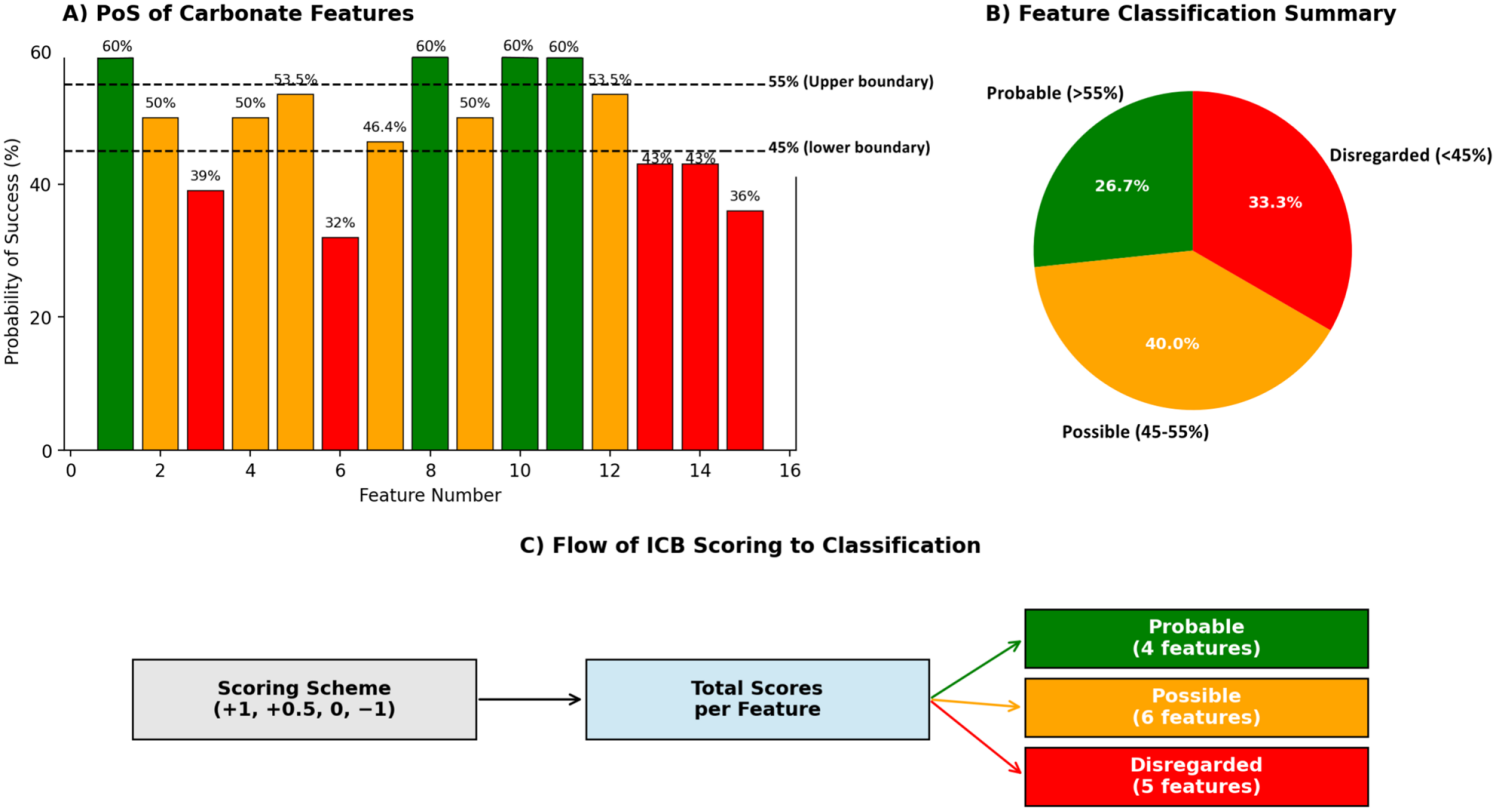
Summary of the Identified carbonate features classification. **a** PoS of carbonate features. **b** Feature classification summary. **c** Flow of ICB scoring classification.

Table 2 summarizes the identification criteria and provides the detailed scoring for each detected carbonate feature. Figure 13 illustrates the spatial distribution of the identified carbonate features on the seismic data based on the proposed criteria, while Fig. 14 presents a summary classification of the evaluated features.

**Table 2:** Detected carbonate features imaged on the seismic data (Fig. 5.12a) and assessed according to application of the identification criteria with quantitative scores applied for each criterion.

	Identification criteria	Feature number							
		1	2	3	4	5	6	7	8
1	Timing relative to Paleolatitude	1	1	1	1	1	1	1	1
2	Syn-sedimentary fault proximity	1	1	1	1	1	1	1	1
3	Short-lived carbonate windows	1	1	1	0.5	0.5	0.5	0.5	0.5
4	Horizon flattening anomalies	1	1	1	1	1	1	1	1
5	Rapid drowning surfaces	0.5	0	0	0.5	0.5	0	0.5	0.5
6	Localized thickening	0.5	0.5	0.5	0.5	0.5	0.5	0.5	0.5
7	Onlap of overburden	0.5	0.5	0.5	0.5	0.5	0.5	0.5	0.5
8	Absence of equivalent structure in overburden	1	0.5	0.5	1	0.5	0.5	0.5	0.5
9	Continuous high-amp reflector cap	0.5	0.5	0.5	0.5	1	0	0.5	1
10	Appropriate seismic facies	0.5	0.5	0	0.5	0.5	0	0.5	0.5
11	Seismic attributes response	1	0.5	0.5	1	1	0.5	0.5	1
12	Critical drowning surfaces	0.5	0.5	0	0	0.5	0	0	0.5
13	Stacking patterns	0.5	0.5	0	0	0	0	0.5	1
14	Coalescing growth patterns	-1	-1	-1	-1	-1	-1	-1	-1
Total score		8.5	7	5.5	7	7.5	4.5	6.5	8.5
Probability of success (%)		60	50	39	50	53.5	32	46.4	60

	Identification criteria	Feature number							
		9	10	11	12	13	14	15	
1	Timing relative to Paleolatitude	1	1	1	1	1	1	1	
2	Syn-sedimentary fault proximity	1	1	1	1	1	1	1	
3	Short-lived carbonate windows	0.5	1	1	1	1	1	1	
4	Horizon flattening anomalies	1	1	1	1	1	1	1	
5	Rapid drowning surfaces	0.5	0.5	0.5	0.5	0.5	0.5	0.5	
6	Localized thickening	1	0.5	0.5	0	0	0	0	
7	Onlap of overburden	0.5	0.5	0.5	0.5	0.5	0.5	0.5	
8	Absence of equivalent structure in overburden	0.5	1	1	1	1	1	0.5	
9	Continuous high-amp reflector cap	0.5	0.5	0.5	0.5	0.5	0.5	0	
10	Appropriate seismic facies	0.5	0.5	0.5	0.5	0	0	0	
11	Seismic attributes response	0.5	0.5	0.5	0.5	0.5	0.5	0.5	
12	Critical drowning surfaces	0	0.5	0.5	0	0	0	0	
13	Stacking patterns	0.5	0	0	0	-1	-1	-1	
14	Coalescing growth patterns	-1	0	0	0	0	0	0	
Total score		7	8.5	8.5	7.5	6	6	5	
Probability of success (%)		50	60	60	53.5	43	43	36

## Discussion

Applying the Burgess et al. (2013)^10^ framework to the Badenian succession of the Pannonian Basin required targeted adaptation to a syn-rift setting characterized by subdued depositional relief and short-lived carbonate growth. Two modifications were essential. First, horizon flattening was integrated into the Basic Geometries step to restore paleobathymetry and remove the effects of structural tilting and differential subsidence. In this tectonically segmented basin, flattening re-established the depositional reference frame and allowed subtle mounds to be distinguished from structural highs. Second, seismic attribute analysis was embedded within the Geophysical Characteristics step. Instantaneous amplitude highlighted impedance contrasts and buildup margins; instantaneous phase improved identification of reflector terminations and internal discontinuities; and pseudo-relief enhanced geomorphic expression on horizon slices. Together, these additions increased interpretive confidence where conventional geometries were ambiguous.

The criteria were further adjusted to reflect Badenian conditions. Within Regional and Stratigraphic considerations, emphasis was placed on syn-sedimentary fault proximity and the short-lived nature of carbonate production. Velocity pull-up and potential-field anomalies were de-emphasized, as they are not diagnostic for shallow-marine Badenian buildups. At the seismic scale, internal chaotic facies and drowning surfaces were incorporated explicitly to capture rapid burial and subsidence. Coupled with a quantitative scoring scheme and PoS ranking, the workflow becomes reproducible and decision-oriented rather than descriptive.

The three seismic attributes proved complementary. Reflection strength consistently mapped the high-impedance contrast at the top-Badenian marl–carbonate contact, but alone it is sensitive to tuning and lateral facies variation. Instantaneous phase, independent of amplitude, was effective at distinguishing continuous bedded facies from disrupted mound interiors, although phase breaks are not uniquely carbonate. Pseudo-relief provided the clearest plan-view morphology, sharpening crest–margin transitions. The most robust interpretations arose from integrating all three attributes and anchoring them to well and core control.

Compared with global analogues, the Pannonian examples are distinct. Miocene buildups in Southeast Asia (e.g., Natuna, Luconia) are large and easily recognized on conventional stacks^34^. In contrast, Badenian buildups are small (tens of meters thick) and short-lived (~ 1.5 Ma), reflecting restricted Paratethyan conditions and syn-rift accommodation. Carboniferous buildups of the Precaspian Basin show strong seismic rims but are deeply buried and imaging limited. Adriatic–Apennine analogues provide the closest comparison, where Miocene buildups nucleate along fault scarps in a tectonically controlled setting^35^. These comparisons highlight that the Pannonian carbonate factories are tectonically focused and rapidly drowned, requiring attribute-driven detection rather than scale-based recognition.

Tectonic and paleoenvironmental controls explain the observed geometries. Syn-sedimentary faulting generated localized accommodation and paleohighs that focused nucleation along fault-block margins. The Badenian transgression enabled growth, whereas subsequent relative sea-level fluctuations and continued subsidence led to drowning, preserved as internal chaotic facies and stratigraphic surfaces. Restricted circulation within the Central Paratethys likely limited lateral progradation, producing isolated edifices rather than extensive platforms. Post-depositional cementation and local dolomitization modified acoustic properties, sometimes enhancing detectability but complicating porosity prediction.

These results have direct exploration implications. High-PoS buildups represent viable stratigraphic-trap candidates where structural closure and effective marl sealing can be demonstrated. The PoS ranking provides a transparent method for prioritizing prospects. Attribute behavior particularly amplitude rims combined with phase disruption shows encouraging correspondence with log-derived porosity trends, suggesting a screening-level role for attribute analysis in reservoir prediction. Because the revised workflow formalizes flattening, attribute integration, and fault proximity within a quantitative framework, it is transferable to other syn-rift carbonate systems.

Limitations remain. With a vertical resolution of ~ 30 m, smaller edifices and thin caps may fall below detection. Attribute responses are non-unique and require geological calibration. Well control is limited to three wells, with only one intersecting a buildup, constraining depositional–diagenetic discrimination and threshold refinement. Future work should include pre-stack acoustic-impedance inversion, AVO analysis, and machine-learning classification of multi-attribute patterns, supported by expanded core and petrographic calibration.

Repositioning horizon flattening within Basic Geometries and formalizing attribute analysis within Geophysical Characteristics, then coupling both to quantitative PoS ranking, enables systematic identification and risking of subtle Badenian buildups in a syn-tectonic basin. The broader lesson is methodological: exploration workflows must be adapted to geological context. A framework designed for large, open-marine platforms can be recalibrated for small, fault-controlled carbonate systems in restricted basins. That adaptability constitutes the principal contribution of this study.

## Conclusion

This study demonstrates that quantitative identification of isolated carbonate buildups is feasible in the structurally complex Pannonian Basin when the Burgess-style workflow is adapted to syn-rift conditions. Integrating horizon flattening within the Basic Geometries step restored paleobathymetry and revealed subtle depositional relief masked by block tilting and differential subsidence. Embedding seismic attributes (instantaneous amplitude, phase, and pseudo-relief) within the Geophysical Characteristics step improved morphological and lithological discrimination where reflector geometry alone was insufficient. Combined with probability-of-success (PoS) ranking, the workflow provides a transparent and reproducible method for distinguishing carbonate buildups from non-carbonate relief.

Application to the dataset identified fifteen candidate features: four classified as high probability, six as moderate, and five as low. High-probability buildups display circular to elongate planforms, rimmed amplitude responses, chaotic-to-parallel internal facies, and consistent proximity to syn-sedimentary faults, consistent with nucleation along fault-block margins. Their spatial distribution reflects the combined influence of tectonic accommodation and restricted Paratethyan conditions, where short-lived Badenian transgressions limited lateral growth and promoted rapid drowning.

In global context, Badenian buildups are smaller and more dispersed than their Southeast Asian or Precaspian counterparts and are not readily identifiable on conventional stacks. Direct application of criteria developed for large, open-marine platforms would misclassify several Pannonian features. Reliable detection requires restoration of depositional reference frames, systematic attribute integration, and quantitative ranking tailored to syn-rift settings.

Exploration implications are direct. High-probability buildups represent viable stratigraphic-trap candidates where structural closure and effective marl sealing are present. The attribute-based ranking provides a practical derisking tool for prospect prioritization. Calibration with Well 1 confirms that the characteristic attribute signatures correspond to carbonate lithology, supporting the workflow’s predictive use at screening scale.

Limitations remain. With an effective vertical resolution of ~ 30 m, smaller edifices may be under-resolved. Attribute responses are non-unique and may mimic volcanic or erosional features. Limited well control constrains regional extrapolation. Future work should incorporate pre-stack impedance inversion, AVO analysis, machine-learning classification of multi-attribute patterns, and expanded stratigraphic calibration.

The principal contribution is methodological. A workflow designed for large, open-marine carbonate platforms has been recalibrated for small, syn-rift carbonate systems in the Central Paratethys. By combining depositional restoration, attribute integration, and probabilistic ranking, the approach offers a transferable framework for identifying and prioritizing carbonate buildups in structurally complex basins.

## Data Availability

The datasets utilized and/or analyzed during the current study are available upon request from the corresponding author.

## Declarations

### Generative AI and AI-assisted technologies in the writing process

During the preparation of this work the authors used ChatGPT to improve the readability of some paragraphs. After using this tool/service, the authors reviewed and edited the content as needed and took full responsibility for the content of the publication.
